# A Scoping Review on the Impact of COVID 19 on Vulnerable Populations: LGBTQ+ Persons, Persons Experiencing Homelessness, and Migrant Farm Workers in the US

**DOI:** 10.26502/aimr.0172

**Published:** 2024-07-29

**Authors:** Donald J. Alcendor, Paul D. Juarez, Aramandla Ramesh, Katherine Y. Brown, Mohammad Tabatabai, Patricia Matthews-Juarez

**Affiliations:** 1 Department of Microbiology, Immunology and Physiology, Center for AIDS Health Disparities Research, School of Medicine, Meharry Medical College, 1005 Dr. D.B. Todd Jr. Blvd., Nashville, TN, 37208-3599, USA.; 2 Center for AIDS Health Disparities Research, Department of Microbiology, Immunology, and Physiology, School of Medicine, Meharry Medical College, 1005 Dr. D.B. Todd Jr. Blvd., Nashville, TN, 37208-3599, USA; 3 Department of Family & Community Medicine, Meharry Medical College, 1005 Dr. D.B. Todd Jr. Blvd., Nashville, TN, 37208-3599, USA; 4 Department of Biochemistry, Cancer Biology, Neuroscience & Pharmacology, Meharry Medical College, 1005 Dr. D.B. Todd Jr. Blvd., Nashville, TN, 37208-3599, USA; 5 School of Graduate Studies and Research, Meharry Medical College, 1005 Dr. D.B. Todd Jr. Blvd., Nashville, TN, 37208-3599, USA

**Keywords:** COVID-19, Primary Care, LGBTQ+, People Experiencing Homelessness, Migrant Farm Workers, Health Disparities

## Abstract

**Purpose:**

The goal of the National Center for Medical Education Development and Research Center (NCMEDR) is to support the education and training of medical students in the care of vulnerable populations. Access to primary care services in the US is fundamental to the health and wellness of all people regardless of their socioeconomic status. LGBQ+ persons, (lesbian, gay, bisexual, transgender, queer, and other sexual and gender minority), Persons Experiencing Homelessness (PEH), and Migrant Farm Workers (MFW) are among the most underserved, marginalized, and socially vulnerable groups in the US. NCMEDR in the Department of Family and Community Medicine at Meharry Medical College was established in part, with funding from the Department of Health and Human Services (DHHS) and the Health Resources and Services Administration (HRSA). NCMEDR was developed to provide educational pathways for transforming medical education and clinical practice in the US by ascertaining whether medical students were being trained to provide primary care, and behavioral health services to LGBTQ+ persons, PEH, and MFW. Here we focus on the impact of the COVID-19 pandemic on these specific populations because they represent marginalized groups that have been heavily impacted by the pandemic, have poor social determinants of health (SDOH), and are more likely to be uninsured, and are less likely to engage primary care providers outside of emergency room care.

**Methods:**

In this study, a scoping literature review was conducted to assess the impact of COVID-19 on primary care of LQBTQ+ persons, PEH, and MFW.

**Results and Discussion:**

The pandemic provided a serious health disparities gap for the defined vulnerable populations under review by the NCMEDR. The pandemic identified the need for transformative measures for clinical practices, medical education, and health care policies required for implementation to improve health care for vulnerable groups. We make recommendations for interventions with defined populations that may influence clinical, environmental health, and SDOH in the COVID era.

**Conclusions:**

The COVID pandemic directed the need for medical schools, health care and social organizations to intervene in new and different ways in vulnerable and marginalized communities. The recommendations provide a model for advancing health equity, access, quality, utilization, care coordination, and treatment.

## Introduction

National Center for Medical Education Development and Research Center (NCMEDR) at Meharry Medical College recognizes the impact of health inequalities among LGBQ+ persons, (lesbian, gay, bisexual, transgender, queer, and other sexual and gender minority), Persons Experiencing Homelessness (PEH), and Migrant Farm Workers (MFW). NCMEDR was established in the Department of Family and Community Medicine at Meharry with funding from the Department of Health and Human Services (DHHS) and the Health Resources and Services Administration (HRSA) under cooperative agreement UH1HP30348 supporting primary care training and enhancement. The goal of the Center was to conduct systems-level, evidence-based research in primary care training to examine the level of teaching in medical schools that included care of vulnerable and marginalized populations. The ultimate goal of the NCMEDR was to provide educational pathways for transforming medical education and clinical practice in the US. To achieve this basic transformation, the NCMEDR sought information about the current medical educational landscape in US schools by conducting research including systematic review, surveys and focus groups on training and care of medically underserved COVID 19 patients.

The Center identified Lesbian, Gay, Bisexual, Transgender, and questioning (LGBTQ), persons experiencing homelessness (PEH), and migrant farmer worker (MFW) as vulnerable populations to examine patterns of inclusion of these populations and their integration in curricular topics, courses, seminars, and webinars. Additionally, the Center planned and did disseminate its research findings from surveys, focus groups, and questionnaires to academic, primary care training, and health care professionals regarding best practices, Evaluation tools for effective interventions were developed to integrate medical education and clinical practices into person-centered culturally competent care with a focus on these vulnerable populations. A Communities of Practice focus group was employed operationalized the concept and practice of exploring and examining the needs of these three populations and translated these research findings into primary care training and to establish clinical training guidelines that will also access the knowledge of medical students about COVID diagnosis, treatment and vaccinations [1, 2].

Historically, socially vulnerable populations including racial/ethnic minorities struggled with risk prevalent conditions such as obesity, hypertension, diabetes, heart disease, kidney disease, lung disease, cancer, and infectious diseases, which also are risk factors for the most severe complications of COVID-19 disease [3, 4]. During the course of the COVID-19 pandemic increased morbidity and mortality was observed among the marginalized and medical underserved populations (LGBTQ+ persons, PEH, and MFW) as well as among racial and ethnic minorities including African Americans, Hispanic/LatinX, and the elderly. It is essential that culturally competent high-quality care for these populations be included in medical school training to achieve health equity.

## Purpose

The primary goal of this study was to review the literature to identify existing barriers to primary health care services experienced by LQBTQ+ persons, PEH and MFW, and to assess the extent to which those barriers were exacerbated by COVID-19. These barriers represent unmet medical needs of socially vulnerable stakeholders that have resulted in increased morbidities and mortality since the onset of COVID-19. A secondary goal of the study was to identify evidence-based, primary care and policy interventions that address the healthcare needs of these three socially vulnerable populations. Identification of how social vulnerabilities have been addressed for LQBTQ+ persons, PEH, and MFW in the era of COVID-19, have important implications for achieving health equity.

LGBTQ populations, persons experiencing homelessness and migrant farm workers are among the most marginalized communities in the US and their experiences during the COVID pandemic requires further investigation [5–7]. LGBTQ communities have experienced increasing depression, anxiety, substance abuse, psychological trauma, discrimination, social isolation, and violence during the COVID-19 pandemic [8–10]. Persons experiencing homelessness are at higher risk for chronic health conditions, increased risk for COVID-19 infection, morbidity, and mortality as well as stigma and marginalization during the COVID pandemic [11–13]. Migrant farm workers have been disproportionately affected by the COVID pandemic. During the pandemic migrant farm workers have a greater need for health services, lack personal protective equipment (PPE), are at greater risk of infection due to COVID-19, lack unemployment benefits due to their immigration status, discrimination, and experiencing environmental, occupational, and social hazards [14–17]. Strategies to improve vaccine education, awareness, and access among these vulnerable populations and to support their engagement with primary care providers for their COVID- related health care needs is essential.

LGBTQ+ persons have endured long-standing mistreatment, discrimination, and in some cases, had their health care needs ignored by the primary care and other health care providers who may possess explicit or implicit biases that lead to their discomfort in addressing them [18–20]. This has led to discomfort and mistrust and led to hesitancy among LGBTQ+ persons in seeking primary care and preventive services [21, 22]. Failure of health care providers to address the primary health care needs of LGBTQ+ persons now serve as a barrier to persons who are LGBTQ+ to seeking and receiving COVID-19 prevention and treatment services.

PEH are at high risk for contracting COVID-19, with greater risks for severe disease because of poorly sustained engagement with the health care system [23]. They are less likely to be vaccinated, have poor access to COVID testing, treatment, and are more likely to have underlying comorbidities that are associated with the more severe complication of COVID-19. They also are more likely to dwell in congregate settings with poor hygiene practices that put them at higher risk for coronavirus transmission [24, 25]. Therefore, PEH requires sustained health care engagement with primary care providers [26, 27].

MFW typically are undocumented front-line workers for agricultural and other businesses (logging, fishing, construction etc.) and are at high risk for severe disease from COVID-19 that lack sustained engagement with a primary care provider or the health care system. They often encounter social barriers when they try to engage with the health care system, such as language, immigration status, work schedule, lack of transportation, child care, etc., and often do not have a personal primary care physician or regular source of care [28, 29]. They also lack easy access to COVID testing and treatment facilities [30–32]. These social barriers have led to increased risk for adverse disease and unchecked comorbidities in this population that need to be addressed simultaneously.

Like other medical education centers with a commitment to training the most up-to-date medical students, the NCMEDR made an intentional decision to examine the impact of the COVID 19 Pandemic on vulnerable populations under review. The questions that the researchers in the Center raised were simple ones: 1) Are medical students being educated to treat the identified vulnerable and marginalized groups with COVID 19; 2) Are medical students in person-centered clinical practices that treat the vulnerable populations experiencing COVID 19? 3) And if so, what are the educational gaps that must be addressed in medical education by examining current evidence-based studies to inform training and clinical transformation in primary care in Tennessee and across the nation.

## Methods

We started by examining the health status of the above-mentioned three vulnerable populations prior to COVID-19 in the US. We examine how primary care services for these three marginalized populations have been affected by COVID and discuss the implications of these strategies for achieving health equity. We also point out the success of approved vaccines but also discuss the need for vaccine education, awareness, and access for these defined vulnerable communities. Our literature search strategy is detailed below.

### Search strategy

An electronic search was conducted in PubMed, OVID, Google Scholar, SCOPUS, and Web of Science databases for articles in English published from January 2020 till October, 2022. The search strategy cross- referenced keywords for COVID-19, medical education (both allopathic and osteopathic), and interventions.

### Inclusion criteria

#### Studies included in this review met the following criteria:

Studies included in this review met the following criteria: (a) assessed knowledge, attitudes, awareness or skills of medical student about COVID-19 diagnosis, treatment and vaccination; (b) were published in English; (c) were conducted with students enrolled in an accredited U.S. medical school or allied health professions schools, and residents undergoing training in primary care psychiatry and gynecology programs. We included vulnerable populations (defined as people who are LGBTQ, experiencing homelessness, or migrant farmworkers) in the search.

#### The MeSH terms used included:

**The MeSH terms used included:** education, medical, undergraduate; violence; discrimination; distress; depression; anxiety; alcohol abuse; hepatitis; HPV; HIV; PrEP; transmission; vaccination.

#### The Boolean terms used for the search of databases were:

The Boolean terms used for the search of databases were: “COVID-19” OR “medical students” OR “medical schools” OR “osteopathic schools” OR “resident physicians” OR “undergraduate programs” OR “students, medical” OR “medicine, osteopathic” OR “education, medical” AND education OR “clinical training” OR didactic OR curriculum OR simulation OR “active learning” OR “problem-based learning” OR lectures OR education OR preceptorship OR “education, professional” OR “clerkships, clinical” OR curriculum OR teaching OR lectures OR screening OR treatment AND “sexual and gender minorities” OR “LGBTQ” OR “vulnerable populations” OR “homeless people” OR “people experiencing homelessness” OR “migrant farmworkers”.

### Exclusion criteria

Articles that addressed COVID-19 impact on non-vulnerable groups, COVID-19 clinical symptoms, morbidity and mortalities were excluded from the review. Studies that were conducted in countries other than the United States, not in English were not included in this review. The following databases were reviewed generating a total of 3,537 total articles: PubMed (2,500), Google Scholar (600), and SCOPUS (437). After 2592 duplicates were removed, 545 articles remained in the search. One hundred and sixty-seven (167) records were further excluded for not meeting the inclusion criteria. After titles and abstracts were reviewed, 310 additional articles were removed, resulting in a total of 68 articles. The full text review resulted in a total of 54 articles that met review criteria.

Three authors (DJA, AR and PDJ) conducted the scoping review. Two study authors (DJA and AR) conducted the initial search of databases, and exclusion of articles that were duplicates, books, book sections, conference proceedings, general, editorial, not written in English, occurred outside of the U.S., or had no abstract available. Three authors independently conducted the quality assessment of research methods (DJA, AR and PDJ). Final quality assessment was done through a consensus process between two authors (DJA and AR).

## Results and Discussion

### Primary care services for the medically underserved in the US during COVID-19.

Primary care services are foundational to a healthy population and any disruptions in primary care services can be devastating for the communities that they serve, especially the most socially vulnerable. The primary health care system supports all occupations engaged in providing health promotion, disease prevention, treatment, rehabilitation and palliative care services, the public health workforce, and those engaged in addressing the social determinants of health [33] Health systems with a strong primary health care component have been shown to result in better health at lower cost [34]. The shortage of primary care doctors to provide care to vulnerable populations Continues to be driven by geography, maldistribution and choice of the kind of patients that they wish to see in their clinical practice.

The roles of the primary healthcare system must continually evolve to meet the changing needs of diverse patient populations. COVID 19 made the choices smaller as the introduction of fatigue and residency became bigger issues for primary care providers on the front line of the pandemic. During the COVID pandemic, patient- centered primary care services have changed to include greater reliance on a team-based care approach that incorporates new and more widespread use of telemedicine, and information technologies that employ artificial intelligence and machine learning approaches [35]. In addition, preventive and public health services have become more important components that address the social determinants of health of patients.

The COVID-19 pandemic has had an overwhelming impact on the primary healthcare workforce and patients. Primary healthcare providers have played a pivotal role in managing patients and implementing pandemic policies. For most underserved populations, primary healthcare providers are the first point of contact with the health system. It is essential that their initial engagement with primary healthcare providers be productive and satisfactory. Too often for socially vulnerable populations, engagement with primary care providers has been discomforting, unpleasant, and discriminatory [36]. These findings support the need for training of primary healthcare students and residents to have experiences in working with diverse patients and communities in order to enhance provider competencies and achieve health equity.

### LGBTQ+ Communities, primary care, and COVID-19

Primary care providers often lack competencies relevant to preventive health and treatment of LGBTQ+ persons on any number of health issues, make discriminatory comments, and/or hold explicit and/or implicit biases that present as barriers to establishing patient-provider trust and willingness of patients to disclose personal and sensitive information [36]. Addressing sensitive topics such as sexual activities and reproductive health in primary care settings requires patient’s disclosure to provider’s highly personal information that can enhance vulnerability to discrimination [37].

A series of examples will be presented of social determinants, that if not addressed, may lead to lack of willingness of LGBTQ+ adolescents from seeking or receiving appropriate healthcare services. First, LGBTQ+ persons experience higher rates of intimate partner violence (IPV) than their heterosexual and/or cisgender peers [38, 39]. When primary care providers are not trained to engage LGBTQ+ survivors on IPV, they may not see them as victims of a crime leaving the adolescent vulnerable to ongoing victimization [39].

A second example is that LGBTQ+ adolescents have been found to experience stigma by pediatric primary care providers who may be unable or unwilling to recognize and address sexual orientation, gender identity, or gender dysphoria with a child [40]. While primary care pediatricians may feel that they have the necessary training to care for LGBTQ+ adolescents, that same sentiment often is not shared by the pediatric patient [40]. Transgender youth, in particular, often are faced with the task of trying to find a primary care provider with the training, competence, and experience who will affirm their gender identity and engage in meaningful dialogue [41–44].

Another example of understanding the importance for cultural awareness in this subgroup of the population is that LGBTQ+ adolescents are at a higher risk of being homeless due to parental rejection and/or physical, emotional and sexual abuse due to their sexual identify. These conditions can contribute to adolescent LGBTQ+ persons being at a greater risk of repeat interpersonal violence victimization and adverse childhood experiences. The lack of meaningful parental involvement in their lives may have indirect effects on LGBTQ+ adolescent health, leading to depression and low self-esteem, and social stigma and isolation. Social isolation is often a barrier for seeking primary healthcare services, which in turn may result in increased risk of severe outcomes due to COVID-19 and other health conditions that require hospitalization.

COVID-19 left an indelible mark on mental health issues of LGBTQ+ people. Sampogna et al. [45] reported problems such as uncertain future, negative emotions, continuing hormone therapy, accessing health facilities and apathy of family members. Sexual minority men from the Asian American and Pacific Islander communities were reported to experience psychological distress during the COVID-19 pandemic [46]. Other problems reported by sexual and gender minorities include loss of job, and lack of money to buy groceries and medications [47]. Compounding these situations are violent incidents against these populations by law-enforcement personnel and discrimination by healthcare providers [48]. These situations call the need for early detection of mental health issues in these vulnerable groups before the issues become full-blown mental disorders.

Nowaskie et al., examined cultural competencies of 127 primary care providers on LGBTQ+ health [49]. The study was performed using a survey that assessed primary care providers’ attitudes, practices, and health knowledge of LGBTQ+ patients. The study revealed that 78% of primary care providers felt comfortable treating LGBTQ+ patients; however, 70.1% felt that they were less informed on specific health needs of LGBTQ+ patients including clinical management, patient referrals, and overall knowledge accuracy of LGBTQ+ issues [49].

Deficiencies in medical knowledge regarding LGBTQ+ patients are a consistent barrier for providing quality healthcare for LGBTQ+ patients expressed by primary care physicians. A survey of medical schools conducted by our team also reaffirms lack of training in US medical schools pertaining to health issues of all three vulnerable populations [38, 39, 40, 50, 51]. Establishing cultural competencies about LGBTQ+ issues should begin at the level of undergraduate medical education by adopting the LGBTQ policy and position statements [52] of the Gay & Lesbian Medical Association (GLMA; currently known as Health Professionals Advancing LGBTQ Equality). There is a great need for LGBTQ+-specific education to increase providers’ awareness, comfortability, and competency in providing LGBTQ+ health care.

Primary care practitioners should participate in LGBTQ+-affirming training focused on improving care for LGBTQ+ by addressing health issues that contribute to the greatest levels of disparities of health outcomes as compared to heterosexual teens. Priority topics should include intimate partner violence (IPV) [53]; working in rural communities [54]; and working in religious and politically conservative, communities. Providing readily accessible information for LGBTQ+ persons to find health care resources such as culturally competent providers is essential. An innovative program known as OutCare Health-a nonprofit 501(c) is a self-sustaining, web-based LGBTQ+ national platform that centralizes local health care resources and culturally- competent providers [49]. This web-based organization conducts needs assessments, identifies resources and primary care providers for the LGBTQ+ community [49]. The platform is focused on improving access to quality care for the LGBTQ+ community by providing the needed resources for the next generation of primary care providers.

### Persons Experiencing Homelessness, primary care, and COVID-19

Prior to the pandemic PEH often suffer from a multitude of health issues such as mental illness, alcohol abuse, drug addiction, heart disease, diabetes, and HIV infection, that are 3–6 times higher than rates found in the general population and are largely not engaged with primary health providers [55]. Before the pandemic PEH were exposed to the elements, had no access to PPE, had underlying comorbidities, and were sheltered in congregate settings. During the COVID pandemic PEH were highly exposed to at risk populations, had no access to PPE, and because of the lack of social distancing and underlying medical conditions were at higher risk for the most severe complications of COVID-19. However, some communities utilized hotels as emergency non-congregate shelters and provided support services to reduce the risk of COVID-19 infection among PEH [56]. COVID-19 exposure from congregate living when compared to the general population has been shown to contribute to an increased frequency of COVID- related hospitalizations for PEH [57]. Substance use for PEH prior to the pandemic was significant however, PEH face specific challenges in the context of the pandemic and substance use in a study by Scarlett et al., showed tobacco (38%−43%), followed by alcohol (26%−34%) increased after the pandemic among PEH [58]. COVID mortality rates in large cities in the US were higher among PEH compared to the general population. A study by Chang et al., showed that in Los Angeles County, PEH had an adult mortality rate that was 20% higher than the general population in the county [59]. PEH include military veterans that prior to the pandemic received transitional housing and healthcare for veterans experiencing homelessness through the Grant and Per Diem (GPD) program and the Homeless Patient Aligned Care Team (HPACT). Due to challenges of the pandemic, veterans, now are experiencing limitations of telehealth for care delivery, primary care provider frustration and burnout due to increased workloads, and funding for PPE [60]. PEH also are disproportionately affected by the opioid crisis leading to a high incidence of disability and mortality [61]. Office-based addiction treatment (OBAT) programs have been to found effective in reducing mortality risk among PEH [62]. Long-term strategies to improve attendance and retention rates are needed. Other outcomes that examine reductions in risk for overdose, medication management, housing stabilization, better physical and mental improvements, and use of pre-exposure prophylaxis (PrEP) for HIV [63].

Prior to the pandemic, IPV was the leading cause of women’s homelessness [64]. Closure of primary services during the pandemic may put PEH at risk of other harms, such as those related to IPV. Barriers that prevent PEH from accessing primary health services include stigma, poor health, problems contacting services, affordability of health services, transportation, medication security, substance addiction, mental health issues, and outstanding warrants [65]. PEH often prioritize immediate basic needs such as food and shelter above their health care needs which become secondary. Delays or gaps in primary care for PEH often result in expensive acute care services provided in the emergency room. As a result, PEH do not receive adequate primary health care services [66]. PEH also tend to have poor access to COVID-19 testing and prevention services, personal protective equipment (PPE), and may find it difficult to follow CDC COVID-19 mitigation practices, such as mask wearing, hand washing, and social distancing, and as a result, are at increased risk for symptomatic disease, hospitalization, and death due to COVID-19.

Sustained linkage to both primary care and housing stabilization services are essential for PEH. Strategies that support the health and wellness of PEH include discharge planning, coordinated care, hospital in-reach, specialized homeless general practice, general practice outreach, and medical recovery centers. The migratory nature of PEH require primary care practitioners to meet this vulnerable population on site, which can be facilitated via mobile health care services. Primary care services for PEH often are supported and operated by nurse practitioners, medical residents, medical and dental students as well as volunteers, outside of the health care system [67].

### Migrant Farm Workers, primary care, and COVID-19

Migrant farm workers (MFW) are a highly vulnerable and marginalized population, who often experience unmanaged comorbidities, and typically have poor access to the primary care health resources. MFW have poor social determinants of health (SODH). Prior to the COVID-19 pandemic, MFW faced a number of challenges including cumulative, health risk factors and behaviors that results in increased risk of exposure to COVID-19 and related adverse health outcomes. A study by Tutor et al., examined MFW in North Carolina that employs more than 78,000 migrant/seasonal farmworkers (MSFWs) annually. They observed unforeseen challenges due to COVID-19 [68]. Even though workers were provided with access to primary care services via the North Carolina Farmworker Health Program or community health centers, COVID presented many challenges, including congregate activities, a need for consistency of COVID-19 related communications, availability of alternate housing, barriers to testing and contact tracing, lack of internet connectivity, and insufficient personal protective equipment [68]. A study conducted by McCoy et al., of 413 MFW found that the majority of participants did not have a primary care physician [23]. Female MFW were more likely to have a personal primary care physician if they had insurance, lived at the study site for more than 5 years and were born in the United States compared to male migrant workers without insurance who were born outside the United States [23]. MFW have transportation issues traveling to primary care clinics in the COVID era. A study by Corwin et al., examined a COVID outbreak among MFW in Iowa that was mitigated via the deployment of a mobile primary clinic [69]. COVID pandemic experiences of MFW and potential inequities have not been well studied and will require further investigation [70].

**Figure 1:**
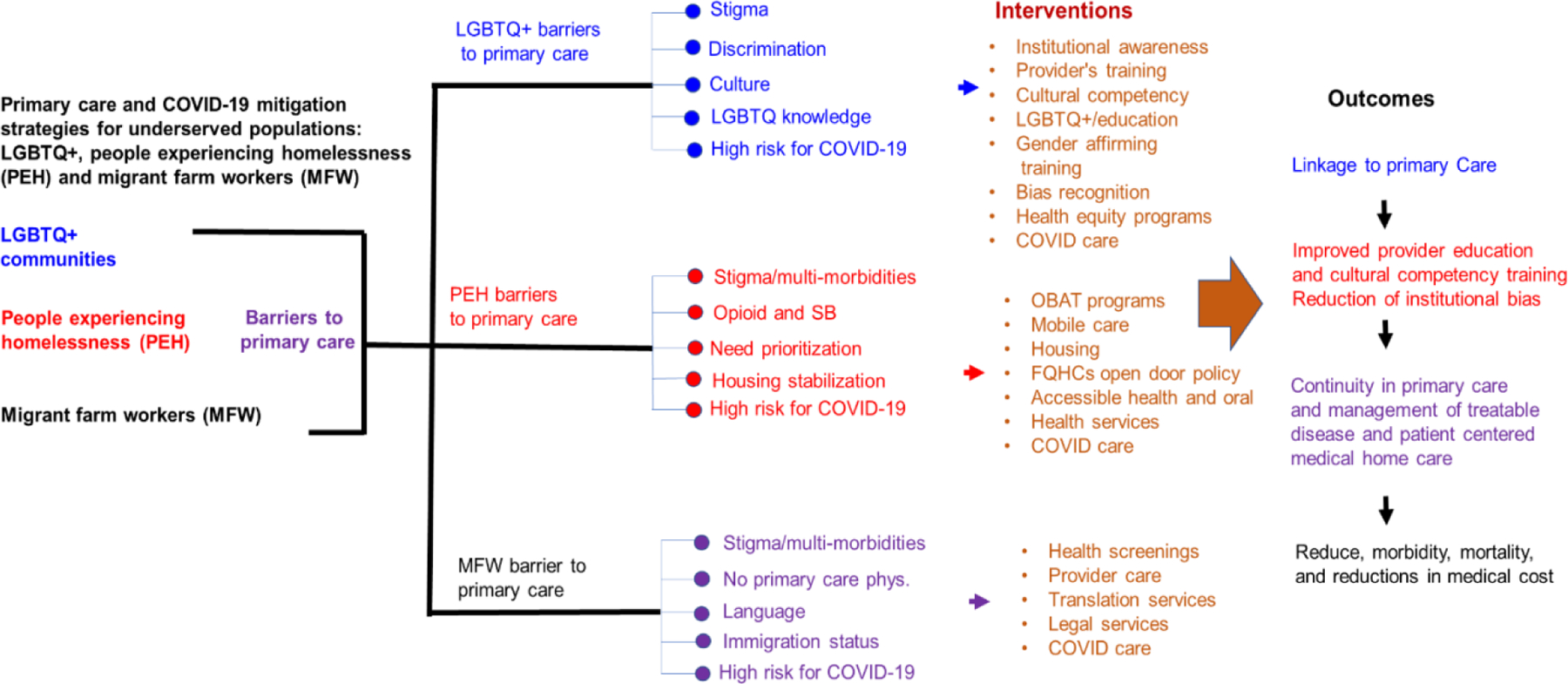
Primary care support and COVID-19 mitigation strategies for LGBTQ+ populations, person experiencing homelessness, and migrant farmworkers.

Barriers to access by vulnerable populations to primary care services and strategy for interventions are examined and presented in the form of a model in Figure 1. Shown are differences and similarities in barriers to primary care access and possible interventions that could lead to improved outcomes for the marginalized populations that include stable linkage to primary care, improved provider education and cultural competencies, reductions in institutional bias and discrimination, patient-entered medical home care, chronic disease management, reduction in morbidity and mortality as well as reductions in medical care cost (Figure 1). All three vulnerable populations examined here experience similar barriers to primary care such as stigma, discrimination, substance abuse, mental health issues, and high risk to COVID-19 infection (Figure 1). Interventions designed to help mitigate these barriers among these populations include education, awareness, stable housing, health services, health screening, and COVID care that includes access to COVID testing and vaccines (Figure 1). Primary outcomes that would benefit all 3 groups include linkage to care, improve provider education, cultural competencies, reduction of institutional bias, continuity in primary care, chronic disease management, patient- centered medical home care, resulting in reduced morbidity, mortality, and reductions in medical cost (Figure 1).

## Conclusions

The COVID-19 pandemic has provided important lessons for strengthening primary healthcare services through better connection between public health, primary care, and secondary care services that better address the needs of socially vulnerable populations. Lessons learned from the pandemic include the importance of a patient- centered team approach that is led by primary care providers to comprehensively address the health and wellness needs of underserved and marginalized communities. The pandemic has helped us identify gaps and barriers to primary care that need to be addressed at scale to effectively change the trajectory of unending health disparities experienced by socially vulnerable populations. Community Health Centers and Migrant Health Centers play an important role in addressing the needs of socially vulnerable populations, as they are the most likely to tailor their services to create sustained access of primary care services. These clinics typically provide COVID-19 testing, vaccines and therapeutics, and access to PPE without cost to the workers. In addition, they often provide mobile care units that go the worksite to minimize barriers for workers who can’t get to scheduled appointments. Timely wellness checks and scheduled screenings for chronic diseases often are made available to workers via mobile clinics in addition to COVID- specific services. However, without sustained support for these approaches and services, lessons learned by the COVID-19 pandemic are likely to be lost.

## References

[1] Matthews-Juarez P, Brown KY, Suara HA. Communities of practice: transforming medical education and clinical practice for vulnerable populations. J. Health Care Poor Underserved 31 (2020): 18–25. 35061606 10.1353/hpu.2020.0135

[2] Brown KYB, Ramesh A, Juarez PD, Innovation in Medical Education: A Communities of Practice Approach. In: Interprofessional Education and Collaborative Practice: International Approaches at Micro, Meso, and Macro Levels. Joosten-Hagye D and Khalili H (Eds). Cognella Academic Publishing, San Diego, CA (2022): 244–253.

[3] Cullen MR, Lemeshow AR, Russo LJ, Disease-specific health disparities: A targeted review focusing on race and ethnicity. Healthcare (Basel) 10 (2022): 603. 35455781 10.3390/healthcare10040603 PMC9025451

[4] Alcendor DJ. Racial disparities-associated COVID-19 mortality among minority populations in the US. J Clin Med 9 (2020): 2442. 32751633 10.3390/jcm9082442 PMC7466083

[5] Salerno JP, Williams ND, Gattamorta KA. LGBTQ populations: Psychologically vulnerable communities in the COVID-19 pandemic. Psychol Trauma 12 (2020): S239–S242. 32551761 10.1037/tra0000837 PMC8093609

[6] Barocas JA, Jacobson KR, Hamer DH. Addressing the COVID-19 pandemic among persons experiencing homelessness: Steps to protect a vulnerable population. J Gen Intern Med 36 (2021): 1416–1417. 33532960 10.1007/s11606-020-06434-5 PMC7852479

[7] Limaye N, Ninesling B, Marcelin F, COVID-19 pandemic response in a migrant farmworker community: Excess mortality, testing access and contact tracing in Immokalee, Florida. Ann Glob Health 88 (2022): 77. 36132278 10.5334/aogh.3859 PMC9461680

[8] Akré ER, Anderson A, Stojanovski K, Depression, anxiety, and alcohol use among LGBTQ+ people during the COVID-19 pandemic. Am J Public Health 111 (2020): 1610–1619. 10.2105/AJPH.2021.306394 PMC8589058 34410817

[9] Gibb JK, DuBois LZ, Williams S, Sexual and gender minority health vulnerabilities during the COVID-19 health crisis. Am J Hum Biol 32 (2020): e23499. 32910838 10.1002/ajhb.23499

[10] Adamson T, Lett E, Glick J, Experiences of violence and discrimination among LGBTQ+ individuals during the COVID-19 pandemic: a global cross-sectional analysis. BMJ Glob Health 7 (2022): e009400. 10.1136/bmjgh-2022-009400 PMC9494011 36130772

[11] Rodriguez NM, Lahey AM, MacNeill JJ, Homelessness during COVID-19: challenges, responses, and lessons learned from homeless service providers in Tippecanoe County, Indiana. BMC Public Health 21 (2021): 1657. 34507565 10.1186/s12889-021-11687-8 PMC8432956

[12] Meehan AA, Thomas I, Horter L, Incidence of COVID-19 among persons experiencing homelessness in the US from January 2020 to November 2021. JAMA Netw Open 5 (2022): e2227248. 10.1001/jamanetworkopen.2022.27248 PMC9389352 35980638

[13] Perri M, Dosani N, Hwang SW. COVID-19 and people experiencing homelessness: challenges and mitigation strategies. CMAJ 192 (2020): E716–E719. 32601252 10.1503/cmaj.200834 PMC7828890

[14] Dudley MJ. Reaching invisible and unprotected workers on farms during the Coronavirus pandemic. J Agromedicine 25 (2020): 427–429. 32894685 10.1080/1059924X.2020.1815625

[15] Castillo F, Mora AM, Kayser GL, Environmental health threats to Latino migrant farmworkers. Annu Rev Public Health 42 (2021): 257–276. 33395542 10.1146/annurev-publhealth-012420-105014 PMC8168948

[16] Boggess B, Bogue HO. The health of U.S. agricultural worker families: A descriptive study of over 790,000 migratory and seasonal agricultural workers and dependents. J Health Care Poor Underserved 27 (2016): 778–792. 27180708 10.1353/hpu.2016.0089

[17] Hu R, Shi L, Lee DC, Access to and disparities in care among migrant and seasonal farm workers (MSFWs) at U.S. health centers. J Health Care Poor Underserved 27 (2016): 1484–1502. 27524780 10.1353/hpu.2016.0107

[18] Morris M, Cooper RL, Ramesh A, Training to reduce LGBTQ-related bias among medical, nursing, and dental students and providers: a systematic review. BMC Med Educ 19 (2019): 325. 31470837 10.1186/s12909-019-1727-3 PMC6716913

[19] Nowaskie DZ, Sowinski JS. Primary Care Providers’ attitudes, practices, and knowledge in treating LGBTQ communities. J Homosex 2019; 66(13):1927–1947. doi: 10.1080/00918369.2018.1519304. 30265839

[20] Heredia D Jr, Pankey TL, Gonzalez CA. LGBTQ-affirmative behavioral health services in primary care. Prim Care 48 (2021): 243–257. 33985702 10.1016/j.pop.2021.02.005

[21] Salerno JP, Williams ND, Gattamorta KA. LGBTQ populations: Psychologically vulnerable communities in the COVID-19 pandemic. Psychol Trauma 12 (2020): S239–S242. 32551761 10.1037/tra0000837 PMC8093609

[22] Akré ER, Anderson A, Stojanovski K, Depression, anxiety, and alcohol use among LGBTQ+ people during the COVID-19 pandemic. Am J Public Health 111 (2021): 1610–1619. 34410817 10.2105/AJPH.2021.306394 PMC8589058

[23] McCoy HV, Williams ML, Atkinson JS, Structural characteristics of migrant farmworkers reporting a relationship with a primary care physician. J Immigr Minor Health 18 (3): 710–714. 26265029 10.1007/s10903-015-0265-2 PMC4752434

[24] Ralli M, Arcangeli A, Ercoli L. Homelessness and COVID-19: Leaving no one behind. Ann Glob Health. 2021; 87 (2021): 11. 33569285 10.5334/aogh.3186 PMC7845474

[25] Self J, Callison K, Goudie A, Medicaid expansion and health services use for adults experiencing homelessness in Arkansas. Health Aff (Millwood) 40 (2021): 91–97. 33400585 10.1377/hlthaff.2019.01812

[26] Paudyal V, Racine M, Hwang SW. COVID-19 vaccination amongst persons experiencing homelessness: practices and learnings from UK, Canada and the US. Public Health 199 (2021): e2–e3. 34548161 10.1016/j.puhe.2021.08.015 PMC8407945

[27] Self JL, Montgomery MP, Toews KA, COVID-19 Homelessness Response Team. Shelter characteristics, infection prevention practices, and universal testing for SARS-CoV-2 at homeless shelters in 7 US urban areas. Am J Public Health 111 (2021): 854–859. 33734836 10.2105/AJPH.2021.306198 PMC8034028

[28] Castillo F, Mora AM, Kayser GL, Environmental health threats to Latino migrant farmworkers. Annu Rev Public Health 42 (2021): 257–276. 33395542 10.1146/annurev-publhealth-012420-105014 PMC8168948

[29] Tutor Marcom R, Freeman Lambar E, Rodman B, Working along the continuum: North Carolina’s collaborative response to COVID-19 for migrant & seasonal farmworkers. J Agromedicine 25 (2020): 409–412. 32921285 10.1080/1059924X.2020.1815621

[30] Lusk JL, Chandra R. Farmer and farm worker illnesses and deaths from COVID-19 and impacts on agricultural output. PLoS One 16 (2021): e0250621. 33909685 10.1371/journal.pone.0250621 PMC8081247

[31] Keeney AJ, Quandt A, Villaseñor MD, Occupational stressors and access to COVID-19 resources among commuting and residential Hispanic/Latinofarmworkers in a US-Mexico border region. Int J Environ Res Public Health 19 (2022): 763. 35055585 10.3390/ijerph19020763 PMC8775392

[32] Castillo F, Mora AM, Kayser GL, Environmental health threats to Latino migrant farmworkers. Annu Rev Public Health 42 (2021): 257–276. 33395542 10.1146/annurev-publhealth-012420-105014 PMC8168948

[33] Dussault G, Kawar R, Castro Lopes S, Building the primary health care workforce of the 21st century - Background paper to the global conference on primary health care: From Alma-Ata towards universal health coverage and the sustainable development goals. Geneva: World Health Organization (2018).

[34] Kringos D The strength of primary care in Europe. Utrecht, Thesis, University of Utrecht, 2012. www.nivel.nl/sites/default/files/bestan-den/Proefschrift-Dionne-Kringos-The-strength-of-primary-care.pdf Accessed Dec 10 (2019).

[35] Sawin G, O’Connor N. Primary care transformation. Prim Care 46 (2019): 549–560. 31655751 10.1016/j.pop.2019.07.006

[36] Rocque R, Leanza Y. A systematic review of patients’ experiences in communicating with primary care physicians: Intercultural encounters and a balance between vulnerability and integrity. PLoS One 10 (2015): e0139577. 26440647 10.1371/journal.pone.0139577 PMC4594916

[37] Kano M, Silva-Bañuelos AR, Sturm R, Stakeholders’ recommendations to improve patient-centered LGBTQ primary care in rural and multicultural practices. J Am Board Fam Med 29 (2016): 156–160. 26769889 10.3122/jabfm.2016.01.150205

[38] Morris M, Cooper RL, Ramesh A, Training to reduce LGBTQ-related bias among medical, nursing, and dental students and providers: a systematic review. BMC Med Educ 19 (2019): 325. 31470837 10.1186/s12909-019-1727-3 PMC6716913

[39] Juarez P, Ramesh A, Cooper RL, A systematic review of the effectiveness of interventions designed to teach medical students to address interpersonal violence across the life course. J. Health Care Poor Underserved 31 (2020): 43–67. 10.1353/hpu.2020.0137 35061608

[40] Bass B, Nagy H. Cultural Competence in the Care of LGBTQ Patients. [Updated 2021 Oct 9]. In: StatPearls [Internet]. Treasure Island (FL): StatPearls Publishing; 2022 Jan-. Available from: https://www.ncbi.nlm.nih.gov/books/NBK563176/33085323

[41] Stern M Perspectives of LGBTQ Youth and pediatricians in the primary care setting: A systematic review. J Prim Care Community Health 12 (2021): 21501327211044357. 34476999 10.1177/21501327211044357 PMC8422807

[42] Lena SM, Wiebe T, Jabbour M. Pediatricians’ knowledge, perceptions, and attitudes towards providing health care for lesbian, gay, and bisexual adolescents. Ann R Coll Physicians Surg Can 35 (2002): 406–410. 12814100

[43] Hadland SE, Yehia BR, Makadon HJ. Caring for lesbian, gay, bisexual, transgender, and questioning youth in inclusive and affirmative environments. Pediatr Clin North Am 63 (2016): 955–969. 27865338 10.1016/j.pcl.2016.07.001 PMC5119916

[44] Cooper RL, Ramesh A, Radix AE, Affirming and inclusive care training for medical students and residents to reducing health disparities experienced by sexual and gender minorities (SGM): A systematic review. Transgender Health (2022). 10.1089/trgh.2021.0148 PMC10387161 37525832

[45] Sampogna G, Ventriglio A, Di Vincenzo M, Mental health and well-being of LGBTQ+ people during the COVID-19 pandemic. Int Rev Psychiatry 34 (2022): 432–438. 36151840 10.1080/09540261.2021.2019686

[46] Lee JJ, Katz DA, Kerani RP, Physical violence and psychological distress among Asian and Pacific Islander sexual minority men in the United States before and during the COVID-19pPandemic. LGBT Health 9 (2022): 418–425. 35766962 10.1089/lgbt.2021.0418 PMC9499447

[47] Adamson T, Lett E, Glick J, Experiences of violence and discrimination among LGBTQ+ individuals during the COVID-19 pandemic: a global cross-sectional analysis. BMJ Glob Health 7 (2022): e009400. 10.1136/bmjgh-2022-009400 PMC9494011 36130772

[48] Levandowski BA, Miller SB, Ran D, Piling it on: Perceived stress and lack of access to resources among US-based LGBTQ+ community members during the COVID-19 pandemic. PLoS One 17 (2022): e0271162. 35802684 10.1371/journal.pone.0271162 PMC9269365

[49] Nowaskie DZ. Development, Implementation, and Effectiveness of a Self-sustaining, Web-Based LGBTQ+ National Platform: A framework for centralizing local health care resources and culturally competent providers. JMIR Form Res 5 (2021): e17913. 34550083 10.2196/17913 PMC8495572

[50] Nair JM, Waad A, Byam S, Barriers to care and root cause analysis of LGBTQ+ patients’ experiences: A qualitative study. Nurs Res 70 (2021): 417–424. 34262007 10.1097/NNR.0000000000000541

[51] Cooper RL, Juarez PD, Morris MC, Recommendations for increasing physician provision of pre-exposure prophylaxis: Implications for medical student training. INQUIRY: The Journal of Health Care Organization, Provision, and Financing 58 (2021a): 469580211017666. 10.1177/00469580211017666 PMC8142521 34027712

[52] GLMA Health Professionals Advancing LGVT Equality (accessed on 5/11/2022 at: https://www.glma.org/_data/n_0001/resources/live/GLMA%20Compendium%20of%20Health%20Profession%20Association%20LGBT%20Policy%20and%20Position%20Statements.pdf).

[53] Bermea AM, Slakoff DC, Goldberg AE. Intimate partner violence in the LGBTQ+ community: experiences, outcomes, and implications for primary care. Prim Care 48 (2021): 329–337. 33985708 10.1016/j.pop.2021.02.006

[54] Shaver J, Sharma A, Stephenson R. Rural primary care providers’ experiences and knowledge regarding LGBTQ health in a midwestern state. J Rural Health 35 (2019): 362–373. 30203423 10.1111/jrh.12322

[55] Top Ten Health Issues Facing Homeless People https://www.homelesshub.ca/blog/top-ten-health-issues-facing-homeless-people accessed August 12 (2021).

[56] Fleming MD, Evans JL, Graham-Squire D, Association of shelter-in-place hotels with health services use among people experiencing homelessness during the COVID-19 pandemic. JAMA Netw Open 5 (2022): e2223891. 35895061 10.1001/jamanetworkopen.2022.23891 PMC9331083

[57] Montgomery MP, Hong K, Clarke KEN, Hospitalizations for COVID-19 among US people experiencing incarceration or homelessness. JAMA Netw Open 5 (2022): e2143407. 35024835 10.1001/jamanetworkopen.2021.43407 PMC8759002

[58] Scarlett H, Melchior M, Davisse-Paturet C, Substance use among residents of homeless shelters during the COVID-19 pandemic: Findings from France. Int J Public Health 67 (2022): 1604684. 36090832 10.3389/ijph.2022.1604684 PMC9452639

[59] Chang AH, Kwon JJ, Shover CL, COVID-19 mortality rates in Los Angeles county among people experiencing homelessness, March 2020-February 2021. Public Health Rep (2022): 333549221115658. 10.1177/00333549221115658 PMC9548447 35989598

[60] Gin JL, Balut MD, Alenkin NR, Responding to COVID-19 while serving veterans experiencing homelessness: The pandemic experiences of healthcare and housing providers. J Prim Care Community Health 13 (2022): 21501319221112585. 35833646 10.1177/21501319221112585 PMC9289898

[61] Ali MM, Sutherland H, Rosenoff E. Comorbid health conditions and treatment utilization among individuals with opioid use disorder experiencing homelessness. Subst Use Misuse 56 (2021): 571–574. 33637031 10.1080/10826084.2021.1884723

[62] Fine DR, Lewis E, Weinstock K, Office-based addiction treatment retention and mortality among people experiencing homelessness. JAMA Netw Open 4 (2021): e210477. 33662132 10.1001/jamanetworkopen.2021.0477 PMC7933994

[63] Doran KM, Boyer AP, Raven MC. Health care for people experiencing homelessness-What outcomes matter? JAMA Netw Open 4 (2021): e213837. 33764419 10.1001/jamanetworkopen.2021.3837

[64] Yakubovich AR, Bartsch A, Metheny N, Housing interventions for women experiencing intimate partner violence: a systematic review. Lancet Public Health 7 (2022): e23–e35. 34838218 10.1016/S2468-2667(21)00234-6

[65] Davies A, Wood LJ. Homeless health care: meeting the challenges of providing primary care. Med J Aust 209 (5): 230–234. 30157413 10.5694/mja17.01264

[66] Albertson S, Murray T, Triboletti J, Implementation of primary care clinical pharmacy services for adults experiencing homelessness. J Am Pharm Assoc 2021; 61 (2021): e80–e84. 10.1016/j.japh.2020.10.012 33160869

[67] Caires AL. Mobile health care for people who are homeless. Creat Nurs 23 (2017): 151–157. 28789733 10.1891/1078-4535.23.3.152

[68] Tutor Marcom R, Freeman Lambar E, Rodman B, Working along the Continuum: North Carolina’s Collaborative Response to COVID-19 for Migrant & Seasonal Farmworkers. J Agromedicine 25 (2020): 409–412. 32921285 10.1080/1059924X.2020.1815621

[69] Corwin C, Sinnwell E, Culp K. A mobile primary care clinic mitigates an early COVID-19 outbreak among migrant farmworkers in Iowa. J Agromedicine 26 (2021): 346–351. 33902394 10.1080/1059924X.2021.1913272

[70] Limaye N, Ninesling B, Marcelin F, COVID-19 pandemic response in a migrant farmworker community: Excess mortality, testing access and contact tracing in Immokalee, Florida. Ann Glob Health 88 (2022): 77. 36132278 10.5334/aogh.3859 PMC9461680

